# The 100 most cited articles in lateral epicondylitis research: A bibliometric analysis

**DOI:** 10.3389/fsurg.2022.913818

**Published:** 2023-02-13

**Authors:** Senbo Zhu, Zeju He, Qing Bi, Li Cao, Haifeng Gu, Qiong Zhang, Fang Chai

**Affiliations:** Department of Orthopedics, Center for Rehabilitation Medicine, Zhejiang Provincial People's Hospital (Affiliated People's Hospital, Hangzhou Medical College), Hangzhou, China

**Keywords:** lateral epicondylitis, bibliometric analysis, citations, tennis elbow, top papers

## Abstract

**Introduction:**

Lateral epicondylitis is a significant clinical problem in orthopaedics. There have been numerous articles written about this. Bibliometric analysis is critical for determining a field's most influential study. We attempt to identify and analyze the top 100 citations in lateral epicondylitis research.

**Materials and methods:**

On December 31, 2021, an electronic search was conducted in the Web of Science Core Collection and the Scopus search engine with no restrictions on publication years, language, or study design. We reviewed each article's title and abstract until the top 100 were documented and evaluated in various ways.

**Results:**

Between 1979 and 2015, the 100 most cited articles were published in 49 journals. The total number of citations ranged from 75 to 508 (mean ± SD, 145.5 ± 90.9), with citation densities ranging from 2.2 to 37.6 citations per year (mean ± SD, 8.7 ± 6.5). The United States is the most productive country, and the 2000s witnessed a surge in lateral epicondylitis research. The year of publication had a moderately positive correlation with citation density.

**Conclusion:**

Our findings offer readers a fresh perspective on historical development hotspot areas of lateral epicondylitis research. Disease progression, diagnosis, and management have always been topics of discussion in articles. PRP-based biological therapy has emerged as a promising area for future research.

## Introduction

Lateral epicondylitis of the humerus (tennis elbow) is a common clinical condition that affects 1%–3% of adults each year (1). It is characterized by pain on the lateral part of the elbow when the patient grasps and lifts objects. According to statistics, lateral epicondylitis affects 10%–50% of tennis players (2). Workers who repeatedly exert excessive exertion for an extended period risk developing lateral epicondylitis (3). According to research, lateral epicondylitis is caused by extensor carpi radialis brevis (ECRB) or common extensor tendon (EDC) tendon degeneration rather than local inflammation (4, 5). On the other hand, the discovery of neuropeptides in the ECRB muscle suggests that neuropathic inflammation may be one of the causes of elbow pain (6, 7). According to some researchers, tennis elbow may be caused by a failure to repair the tendon and local vascular damage. Regular tendon repair can be hampered by subsequent injury, whereas damaged tendons obstruct tendon repair (8). Several studies on lateral epicondylitis have been published in articles and reviews over the last few decades, covering topics such as prevalence, pathology, injury mechanism, and treatment methods (9–11).

In recent years, bibliometric analysis has emerged in the orthopaedics literature, with various bibliometric articles ranging from rotator cuff tears to total joint arthroplasty (12–15). The citation number (the total number of times other works have cited an article since it was published) and the citation density (the citation number divided by the years since its first publication date) are commonly used to measure an article's influence on its respective specialty. While not thoroughly assessing the quality of a study, the two parameters mentioned above represent a portion of the quality of the articles published. To our knowledge, no other authors have conducted a bibliometric analysis of lateral epicondylitis. This research aimed to identify hotspots and trends in lateral epicondylitis research.

## Materials and methods

### Process design

**Figure 1:**
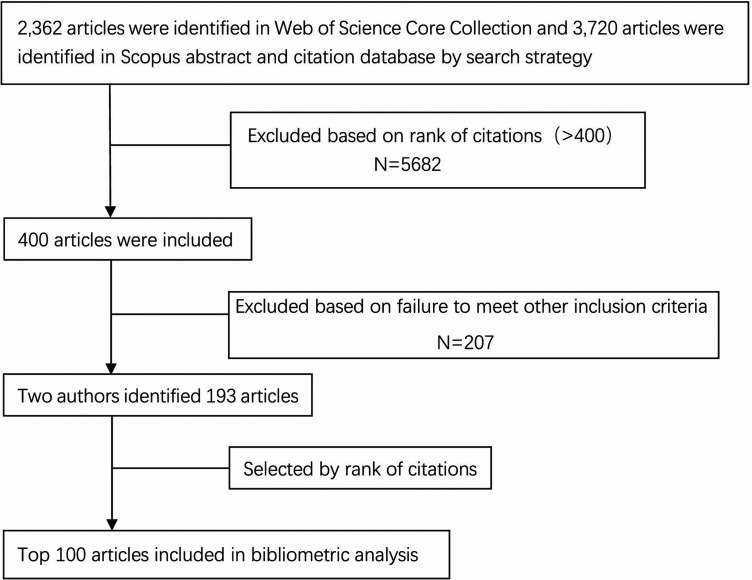
Flowchart illustrating the articles selection process, detailed procedures for screening and admission Note: A total of 2,362 and 3,720 articles were found in the Web of Science Core Collection and Scopus. The top 400 articles were selected according to citation frequency. Two reviewers selected 193 articles based on inclusion and exclusion criteria. The selected articles were ranked in order of citation frequency from high to low, and 100 articles with the highest citation frequency were selected for further investigation.

The trend analysis was carried out on December 31, 2021, using the Web of Science Core Collection (Clarivate Analytics, Toronto, Canada) and the Scopus abstract and citation database (Elsevier, Amsterdam, The Netherlands). The following was the search strategy: Search for “lateral epicondylitis” in the NCBI's MeSH database. Advanced search for all search terms listed below: “Elbow, Tennis” OR “Elbows, Tennis” OR “tennis elbow” OR “Lateral Epicondylitis” OR “Epicondylitides, Lateral” OR “Epicondylitis, Lateral” OR “Lateral Epicondylitides” OR “Epicondylitis, Lateral Humeral” OR “Epicondylitides, Lateral Humeral” OR “Humeral Epicondylitides, Lateral” OR “Humeral Epicondylitis, Lateral” OR “Lateral Humeral Epicondylitides” OR “Lateral Humeral Epicondylitis” (no quotation marks) was taken using Web of Science Core Collection and the Scopus abstract and citation database search engines. Articles from all years and journals were included. A total of 2,362 articles were found in descending order based on the times they were cited in the Web of Science Core Collection. A total of 3,720 articles were found in descending order based on the times they were cited in the Scopus abstract and citation database. The top 400 kinds of literature were chosen based on citation rank; articles ranked higher than 400 were removed. Based on inclusion and exclusion criteria, two reviewers independently conducted the following reviews: Articles that meet the requirements were exported to an Excel spreadsheet. Each reviewer selected an article by reading the full text. If two researchers disagreed, the team will discuss it until they reached an agreement. Articles chosen by the two reviewers were arranged in descending order of citation frequency, and 100 articles with the highest citation frequency were selected for further investigation (Figure 1).

### Inclusion and exclusion criteria

The following criteria for inclusion are (1) basic scientific research and clinical research on tennis elbow; (2) studies on diagnosis, treatment, prognosis, or prevention of tennis elbow; (3) original articles, reviews, case reports, editorials, clinical trials, and other types of papers. The exclusion criteria were as follows: (1) irrelevant to tennis elbow; (2) tennis elbow was not the primary focus of the subject; (3) the original text and other information could not be found.

For each record, the following objective data were extracted: the date of publication, the number of times cited, the number of years since publication, the citation density (defined as the average number of citations per year since publication), the source journal, the primary institution, and the country of origin (according to the address of the corresponding author). Instead of online preview publication, the date was recorded as the month and year the research was formally published in a journal. The publication date was recorded as the first of the month to calculate citation density.

For each article, the following subjective characteristics were also recorded: These details include the article type (clinical research or basic research), as well as the article subtype (clinical study subtypes: a review article, technique article, meta-analysis or systematic review, case-control, case series study, retrospective cohort study, prospective cohort study, nonrandomized controlled trial, randomized controlled trial, diagnostic study, expert opinion; preliminary study subtypes: biomechanical study, anatomic study, review article, *in vitro* study, animal study). Furthermore, the level of evidence for each clinical article was assessed using the classification system proposed by Wright et al. in The Journal of Joint and Bone Surgery (16). Two authors independently reviewed each article for these subjective characteristics; if there was a disagreement, a third reviewer was brought in.

### Statistics

The bibliometrics software Vosviewer_1.6.17, Microsoft Office Excel (Home and Student Edition 2021), GraphPad Prism 8 (GraphPad Software Inc, CA, United States), and IBM SPSS Statistics 26.0 were used for data collection and statistical analysis. Vosviewer_1.6.17 was employed to extract essential information, create a network map of high-frequency keywords, and perform cluster analysis. The Shapiro-Wilk test was used to determine the normality of the distribution of individual variables. Data from a normal distribution are expressed as mean ± standard deviation. A one-way analysis of variance (ANOVA) was used when comparing mean values, and the Student's t-test was used when necessary. The Pearson test was used to examine the relationship between variables, and a difference of *P* < 0.05 was considered significant.

## Results

The number of citations ranged from 75 to 508 citations (mean ± SD, 145.5 ± 90.9) for the 100 most cited articles in lateral epicondylitis research, with citation densities ranging from 2.2 to 37.6 citations per year (mean ± SD, 8.7 ± 6.5) (Supplementary Table S1).

### Publication trend

**Figure 2:**
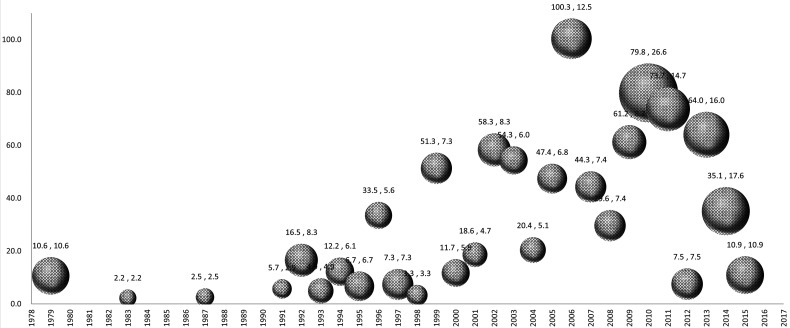
Bubble plot of publication year and mean citation density. Note: The X-axis represents the time range of occurrence of the top 100 cited articles, the Y-axis represents the total citation density, and the bubble size represents the average citation density of each article per year. In the data label, the former is the Y-axis value and the latter is the bubble size value.

The top 100 articles were published between 1979 and 2015, with most appearing in the 2000s (61 articles). The findings revealed a positive relationship between citation density and year of publication (*r *= +0.561, *P* < 0.001) (Figure 2).

### Country distribution

**Figure 3:**
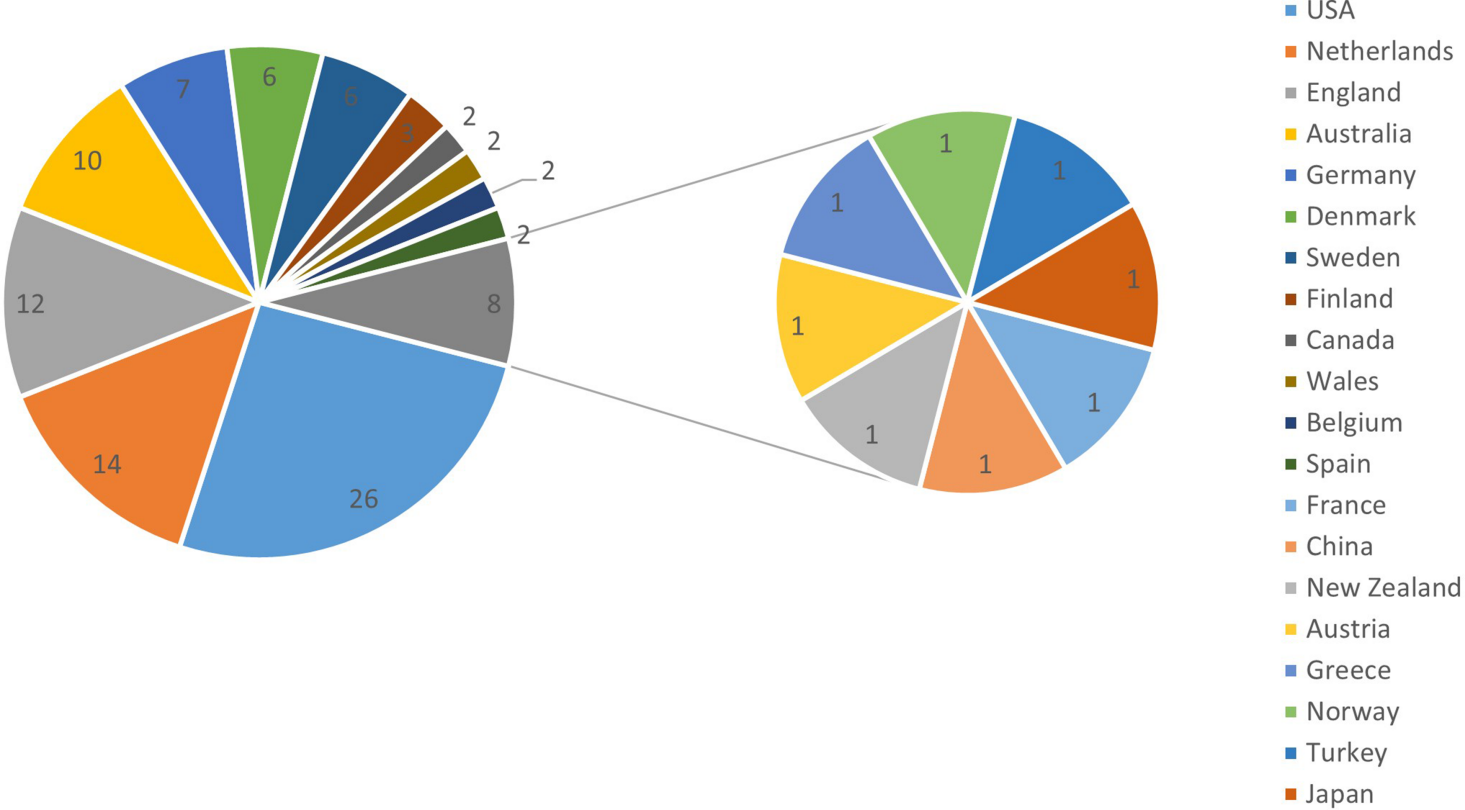
National distribution of the top 100 articles cited in tennis elbow. Note: the numbers in the pie chart represent the number of articles, and the different colors correspond to the countries or regions on the right.

The top 100 most cited articles came from 20 different countries and regions. The United States, the Netherlands, the United Kingdom, Australia, Germany, Denmark, Sweden, Finland, Canada, Wales, Belgium, Spain, France, China, New Zealand, Austria, Greece, Norway, Turkey, and Japan were the most common locations for lateral epicondylitis research articles to be published (Figure 3). The United States has been the leading country to publish articles on this subject.

### Institution distribution and journal of publication

The University of Queensland's Institute for Research was the top contributing research center, accounting for seven top-cited articles. The American Journal of Sports Medicine was the most common place for publication, accounting for 13% of the top-cited articles.

The top 100 cited articles in tennis elbow research were published in 49 journals (Supplementary Table S2). The top three source journals on lateral epicondylitis (American Journal of Sports Medicine, British Journal of Sports Medicine, and Journal of Bone and Joint Surgery-American Volume) published 30 articles on the condition, accounting for 30% of all articles included in this study. Most articles were published in the American Journal of Sports Medicine (13), followed by the British Journal of Sports Medicine and the Journal of Bone and Joint Surgery-American Volume. The Lancet articles had the highest average citation density (28.8), followed by the American Journal of Epidemiology (21.6) and the Journal of the American Medical Association (18.7).

### The author contributions and article type

**Figure 4:**
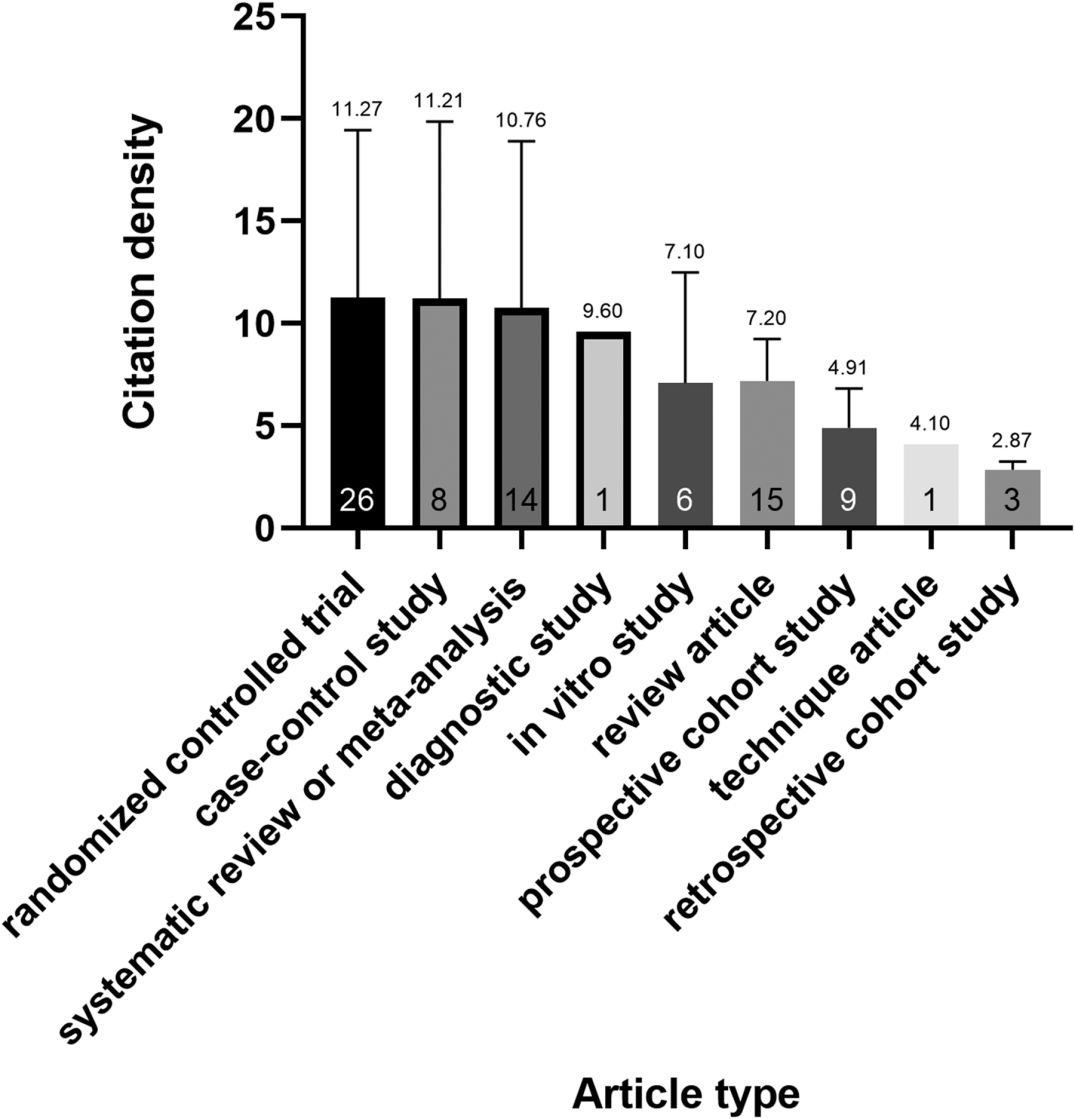
Mean citation density based on the article type. Note: The number at the bottom of the column represents the number of articles. there was no significant difference between article types.

**Figure 5:**
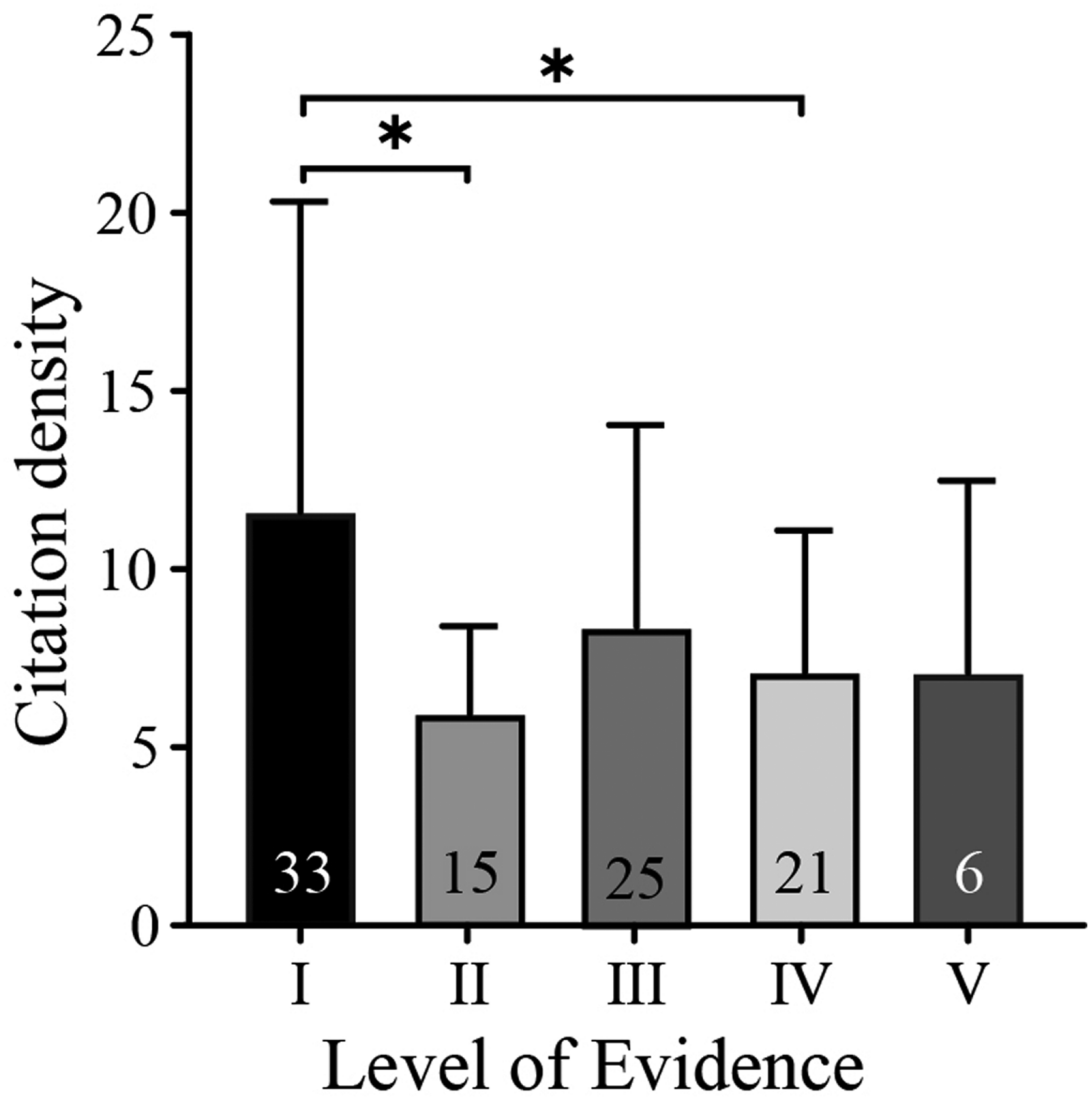
Mean citation density based on the level of evidence. Note: The number at the bottom of the column represents the number of articles. There was a significant difference between levels I and II and levels I and IV, * means *p* < 0.05.

In the top 100 most cited articles, Smidt, N was listed as the first author five times. With 508 citations, the most cited publication was a study by Mishra et al. Most articles were clinical research, with only 6% being basic science. The most common clinical article subtypes were randomized controlled trial (*n* = 26; mean ± SD number of citation density, 11.27 ± 8.18), case-control study (*n* = 8; mean ± SD, 11.21 ± 8.66), systematic review or meta-analysis (*n* = 14; mean ± SD, 10.76 ± 8.13), diagnostic study (*n* = 1; 9.60), *in vitro* study (*n* = 6; mean ± SD, 7.10 ± 5.39), review article (*n* = 15; mean ± SD, 7.20 ± 2.03), prospective cohort study (*n* = 9; mean ± SD, 4.91 ± 1.92), technique article (*n* = 1; 4.10), retrospective cohort study (*n* = 3; mean ± SD, 2.87 ± 0.40) (Figure 4). The one-way ANOVA (*P* = 0.1354) showed no significant difference between article types. According to the criteria established by Wright et al. in The Journal of Bone and Joint Surgery, level I includes randomized controlled trial; level II includes prospective cohort study and poor-quality randomized controlled trial; level III includes case-control study and a retrospective cohort study; level IV includes case series. Nearly one-third (33 articles) of the 94 clinical articles were classified as level I evidence (*n* = 33; mean ± SD number of citation density, 11.62 ± 8.70), level II (*n* = 15; mean ± SD, 5.95 ± 2.45), level III (*n* = 25; mean ± SD, 8.38 ± 5.67), level IV (*n* = 21; mean ± SD, 7.12 ± 3.97) and level V (*n* = 6; mean ± SD, 7.10 ± 5.39) (Figure 5). There was a significant difference between levels I and II and levels I and IV (*P* < 0.05).

### Keywords analysis and research interest

**Figure 6:**
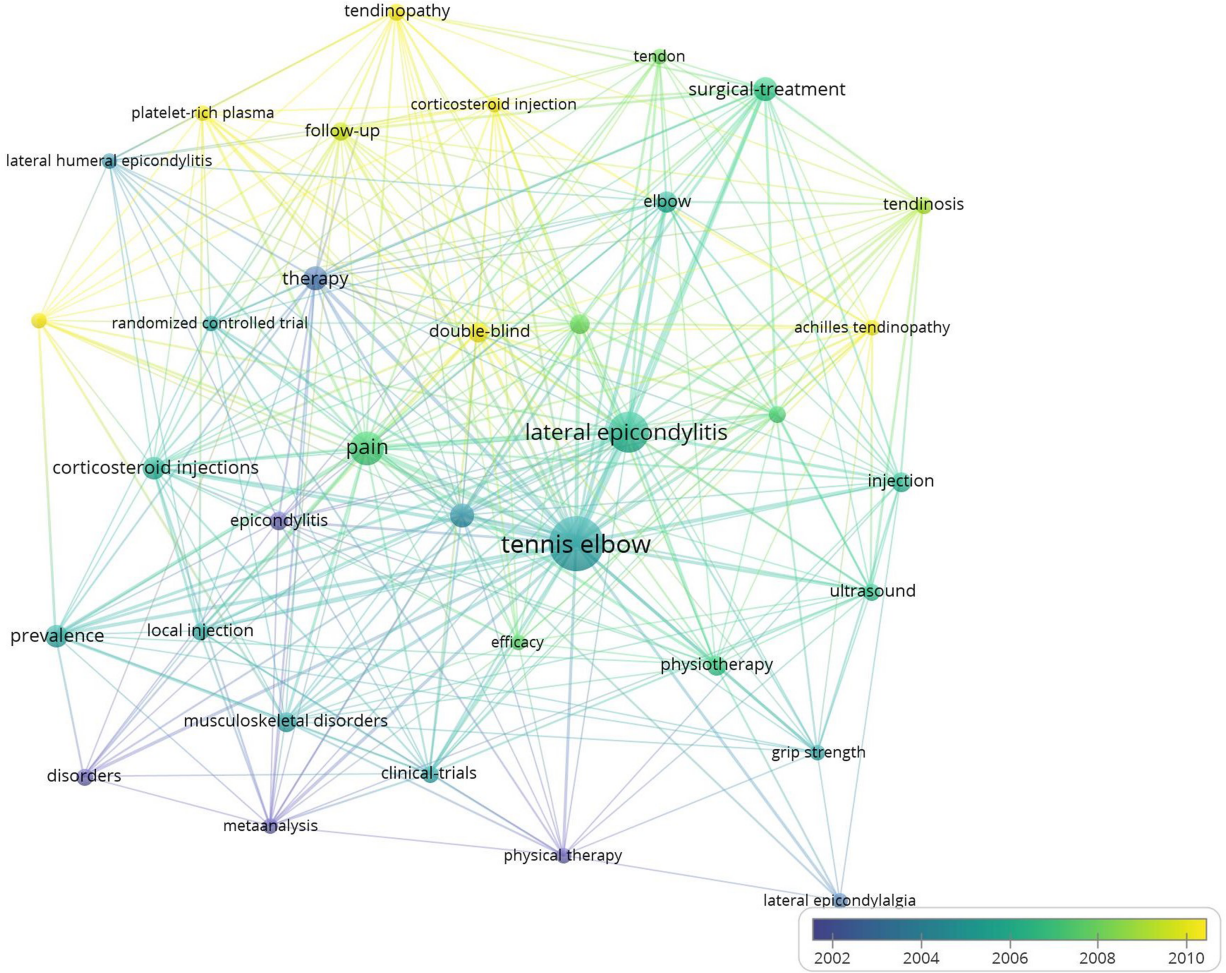
Network visualization map showing cluster analysis of tennis elbow related keywords. Note: The size of each circle represents the number of occurrences, and the more times, the larger the circle. In cluster analysis, different colors indicate different years.

The keywords of each article are analyzed using network analysis, as shown in the diagram (Figure 6). Only keywords that appeared more than five times are counted, and there are a total of 35 keywords; the color change from purple to yellow indicates the period from 2002 to 2010. The visual figure, tennis elbow, lateral epicondylitis, and pain had the highest degree of centrality, but by 2010, the focus had shifted to platelet-rich plasma and corticosteroid injection.

## Discussion

Bibliometric studies use a variety of methods to assess the importance of publications. One way is to use the number of citations of a given article to determine its impact on the literature. While this is not a perfect system, as even poorly done research can be frequently cited, it is still widely used to determine the impact of journals and researchers. Bibliometric studies are particularly useful when evaluating a topic with many publications. One such topic is the study of tennis elbow, which has attracted increasing interest from sports medicine surgeons and their patients. There are thousands of articles on tennis elbow in the medical literature, reflecting the intense interest in tennis elbow. One problem with such a large number of related articles is that it is almost impossible for even experts in the field, let al.one novices, to keep up with all the literature or even to identify the most important ones they need to be familiar with. For this reason, we believe that a bibliometric analysis of the tennis elbow literature is long overdue; To the best of our knowledge, no such analysis exists in the literature.

Based on our search, we found a significant increase in publications related to lateral epicondylitis over the last several decades, from 4 papers published in 1980 to 88 papers published in 2021. The current study examined the top 100 most cited articles between 1979 and 2015, and several conclusions were drawn based on our findings. First, lateral epicondylitis, also known as tennis elbow, has been a constant source of concern, with numerous articles appearing in the 21st century. Second, the disease course, anatomy, physical and accessory examination methods, and various surgical or conservative treatment methods are hotspots in this field of study. Many issues remain unresolved in the diagnosis, treatment, recurrence, and prognosis of lateral epicondylitis.

As one might expect from classical bibliometric analysis theory, papers published a few years earlier have had more possibility to accumulate citations (17). The citation density, defined as the average number of citations per year since publication, helps to compensate for this. The citation density of this list's most recently published articles is high. There is a statistically significant and moderately positive correlation between the citation density and the year of publication (*P* < 0.001). When the number of citations for recently published articles is considered, the most popular list must be cited more than previous articles. This discovery is, of course, intuitive. The level I and IV/V studies each account for roughly one-third of the most cited articles, indicating that low-level evidence publications in this research field are highly cited. The article publication dates in our analysis are roughly bell-shaped, with a peak around 2003. This period could be associated with the peak epidemic of lateral epicondylitis research. In terms of distribution by country/region, the United States and the Netherlands are the two leading countries. Dr. Smidt N's group published the most articles (five) between 2002 and 2006, focusing on conservative treatment of lateral epicondylitis (18–22). According to JCR, the top ten journals publishing articles on lateral epicondylitis were mostly medical journals, with only one sports science journal among the top ten. Most of these top ten journals were classified as SCI quantile 1 or 2, giving them more credibility for future researchers to study and cite.

The articles on our most-cited list provide insights into the history of lateral epicondylitis research and may aid in documenting its evolutionary course over time. Dr. Mishra's top-cited article was one of the most influential articles that sought platelet-rich plasma (PRP) treatment for chronic elbow tendinosis (23). The first, from 1973, was published in The Journal of Bone and Joint Surgery-American volume describing the effect of surgery on tennis elbow at the time (24). Tennis elbow surgery resulted in an overall improvement rate of 97.7%, with 85.2% of patients returning to complete activities, including vigorous exercise. As the disease became more widely known, various methods for diagnosing and treating it appeared and were reported in the early 1990s (5, 8, 25, 26). Local corticosteroid injection and low-energy extracorporeal shock became popular tennis elbow treatment methods in the late 1990s (27–30). Dr. Baker, CL et al. were the first of 100 articles to include an arthroscopic release in alternative management (31). More researchers focused on biological methods in the 21st century, as injection of PRP (platelet-rich plasma) and botulinum toxin became popular in more extensive multicenter randomized trials by Dr. Thanasas et al. Dr. Peerbooms et al. and Dr. Wong et al. (32–34). However, according to Dr. Robert-systematic Jan's review, there is strong evidence that PRP injection is ineffective in treating chronic lateral elbow tendinopathy (35). Workload and strain have long been studied for their potential link to morbidity, but agreement among studies has been elusive.

There are a few limitations to this study. Firstly, more recent and influential articles may not appear in the top 100 most cited lists because the more recent publications may have a problem accumulating references due to a lack of time. Secondly, self-citation may distort the number of citations and the citation density index, overestimating the impact of a specific publication in a particular field. A more precise bibliometric analysis method is required to take this into account.

## Data Availability

The datasets presented in this study can be found in online repositories. The names of the repository/repositories and accession number(s) can be found in the article/**Supplementary Material**.
