# Supplementary material for: The 100 most cited articles in lateral epicondylitis research: A bibliometric analysis
Source: Front Surg. 2023 Feb 13;9:913818. doi: 10.3389/fsurg.2022.913818 (PMC9968860; doi:10.3389/fsurg.2022.913818)
Supplement: Supplementary file 1 [file Table1.docx]

| Table 1. The Top 100 Cited Articles on Tennis elbow | | | | | | | |
| --- | --- | --- | --- | --- | --- | --- | --- |
| Rank | Article Title | Citation Density | Citation | All Authors | Journal Abbreviation | Publication Year、 Volume and Pages |  |
| 1 | Efficacy and safety of corticosteroid injections and other injections for management of tendinopathy: a systematic review of randomised controlled trials | 37.6 | 451 | Coombes, BK; Bisset, L; Vicenzino, B | LANCET | 2010 Nov 20;376(9754):1751-67. |  |
| 2 | Positive Effect of an Autologous Platelet Concentrate in Lateral Epicondylitis in a Double-Blind Randomized Controlled Trial Platelet-Rich Plasma Versus Corticosteroid Injection With a 1-Year Follow-up | 33.3 | 400 | Peerbooms, JC; Sluimer, J; Bruijn, DJ; Gosens, T | AM J SPORT MED | 2010 Feb;38(2):255-62. |  |
| 3 | Treatment of chronic elbow tendinosis with buffered platelet-rich plasma | 31.8 | 508 | Mishra, A; Pavelko, T | AM J SPORT MED | 2006 Nov;34(11):1774-8. |  |
| 4 | Ongoing Positive Effect of Platelet-Rich Plasma Versus Corticosteroid Injection in Lateral Epicondylitis A Double-Blind Randomized Controlled Trial With 2-year Follow-up | 26.8 | 295 | Gosens, T; Peerbooms, JC; van Laar, W; den Oudsten, BL | AM J SPORT MED | 2011 Jun;39(6):1200-8. |  |
| 5 | Efficacy of Platelet-Rich Plasma for Chronic Tennis Elbow A Double-Blind, Prospective, Multicenter, Randomized Controlled Trial of 230 Patients | 23.0 | 184 | Mishra, AK; Skrepnik, NV; Edwards, SG; Jones, GL; Sampson, S; Vermillion, DA; Ramsey, ML; Karli, DC; Rettig, AC | AM J SPORT MED | 2014 Feb;42(2):463-71. |  |
| 6 | Prevalence and determinants of lateral and medial epicondylitis: A population study | 21.6 | 345 | Shiri, R; Viikari-Juntura, E; Varonen, H; Heliovaara, M | AM J EPIDEMIOL | 2006 Dec 1;164(11):1065-74. |  |
| 7 | Treatment of Lateral Epicondylitis With Platelet-Rich Plasma, Glucocorticoid, or Saline A Randomized, Double-Blind, Placebo-Controlled Trial | 20.8 | 187 | Krogh, TP; Fredberg, U; Stengaard-Pedersen, K; Christensen, R; Jensen, P; Ellingsen, T | AM J SPORT MED | 2013 Mar;41(3):625-35. |  |
| 8 | Corticosteroid injections, physiotherapy, or a wait-and-see policy for lateral epicondylitis: a randomised controlled trial | 20.1 | 401 | Smidt, N; van der Windt, DAWM; Assendelft, WJJ; Deville, WLJM; Korthals-de Bos, IBC; Bouter, LM | LANCET | 2002 Feb 23;359(9307):657-62. |  |
| 9 | Effect of Corticosteroid Injection, Physiotherapy, or Both on Clinical Outcomes in Patients With Unilateral Lateral Epicondylalgia A Randomized Controlled Trial | 18.7 | 168 | Coombes, BK; Bisset, L; Brooks, P; Khan, A; Vicenzino, B | JAMA-J AM MED ASSOC | 2013 Feb 6;309(5):461-9. |  |
| 10 | Tendinosis of the elbow (tennis elbow) - Clinical features and findings of histological, immunohistochemical, and electron microscopy studies | 17.8 | 410 | Kraushaar, BS; Nirschl, RP | J BONE JOINT SURG AM | 1999 Feb;81(2):259-78. |  |
| 11 | Platelet-Rich Plasma Versus Autologous Whole Blood for the Treatment of Chronic Lateral Elbow Epicondylitis A Randomized Controlled Clinical Trial | 17.4 | 191 | Thanasas, C; Papadimitriou, G; Charalambidis, C; Paraskevopoulos, I; Papanikolaou, A | AM J SPORT MED | 2011 Oct;39(10):2130-4. |  |
| 12 | Mobilisation with movement and exercise, corticosteroid injection, or wait and see for tennis elbow: randomised trial | 16.8 | 269 | Bisset, L; Beller, E; Jull, G; Brooks, P; Darnell, R; Vicenzino, B | BMJ-BRIT MED J | 2006 Nov 4;333(7575):939. |  |
| 13 | Growth factor-based therapies provide additional benefit beyond physical therapy in resistant elbow tendinopathy: a prospective, single-blind, randomised trial of autologous blood injections versus platelet-rich plasma injections | 14.6 | 161 | Creaney, L; Wallace, A; Curtis, M; Connell, D | BRIT J SPORT MED | 2011 Sep;45(12):966-71. |  |
| 14 | Comparative Effectiveness of Injection Therapies in Lateral Epicondylitis A Systematic Review and Network Meta-analysis of Randomized Controlled Trials | 13.3 | 120 | Krogh, TP; Bartels, EM; Ellingsen, T; Stengaard-Pedersen, K; Buchbinder, R; Fredberg, U; Bliddal, H; Christensen, R | AM J SPORT MED | 2013 Jun;41(6):1435-46. |  |
| 15 | Strong evidence against platelet-rich plasma injections for chronic lateral epicondylar tendinopathy: a systematic review | 12.1 | 97 | de Vos, RJ; Windt, J; Weir, A | BRIT J SPORT MED | 2014 Jun;48(12):952-6. |  |
| 16 | A systematic review and meta-analysis of clinical trials on physical interventions for lateral epicondylalgia | 12.1 | 206 | Bisset, L; Paungmali, A; Vicenzino, B; Beller, E | BRIT J SPORT MED | 2005 Jul;39(7):411-22; discussion 411-22. |  |
| 17 | Real-Time Sonoelastography of Lateral Epicondylitis: Comparison of Findings Between Patients and Healthy Volunteers | 11.8 | 153 | De Zordo, T; Lill, SR; Fink, C; Feuchtner, GM; Jaschke, W; Bellmann-Weiler, R; Klauser, AS | AM J ROENTGENOL | 2009 Jul;193(1):180-5. |  |
| 18 | Lateral epicondylitis A REVIEW OF PATHOLOGY AND MANAGEMENT | 11.2 | 101 | Ahmad, Z; Siddiqui, N; Malik, SS; Abdus-Samee, M; Tytherleigh-Strong, G; Rushton, N | BONE JOINT J | 2013 Sep;95-B(9):1158-64. |  |
| 19 | The Epidemiology and Health Care Burden of Tennis Elbow A Population-Based Study | 10.9 | 76 | Sanders, TL; Kremers, H; Bryan, AJ; Ransom, JE; Smith, J; Morrey, BF | AM J SPORT MED | 2015 May;43(5):1066-71. |  |
| 20 | TENNIS ELBOW - SURGICAL TREATMENT OF LATERAL EPICONDYLITIS | 10.6 | 456 | NIRSCHL, RP; PETTRONE, FA | J BONE JOINT SURG AM | 1979 Sep;61(6A):832-9. |  |
| 21 | Chronic tendinopathy: effectiveness of eccentric exercise | 10.1 | 151 | Woodley, BL; Newsham-West, RJ; Baxter, GD | BRIT J SPORT MED | 2007 Apr;41(4):188-98; discussion 199. |  |
| 22 | A systematic review with procedural assessments and meta-analysis of Low Level Laser Therapy in lateral elbow tendinopathy (tennis elbow) | 9.8 | 137 | Bjordal, JM; Lopes-Martins, RAB; Joensen, J; Couppe, C; Ljunggren, AE; Stergioulas, A; Johnson, MI | BMC MUSCULOSKEL DIS | 2008 May 29;9:75. |  |
| 23 | ELBOW TENDONOSIS TENNIS ELBOW | 9.6 | 289 | NIRSCHL, RP | CLIN SPORT MED | 1992 Oct;11(4):851-70. |  |
| 24 | Validation of the patient-rated tennis elbow evaluation questionnaire | 9.6 | 144 | Rompe, JD; Overend, TJ; MacDermid, JC | J HAND THER | 2007 Jan-Mar;20(1):3-10; quiz 11. |  |
| 25 | Widespread Mechanical Pain Hypersensitivity as Sign of Central Sensitization in Unilateral Epicondylalgia A Blinded, Controlled Study | 9.4 | 122 | Fernandez-Carnero, J; Fernandez-de-las-Penas, C; de la Llave-Rincon, AI; Ge, HY; Arendt-Nielsen, L | CLIN J PAIN | 2009 Sep;25(7):555-61. |  |
| 26 | Associations between work-related factors and specific disorders at the elbow: a systematic literature review | 9.3 | 121 | van Rijn, RM; Huisstede, BMA; Koes, BW; Burdorf, A | RHEUMATOLOGY | 2009 May;48(5):528-36. |  |
| 27 | Ultrasound therapy for musculoskeletal disorders: A systematic review | 9.3 | 214 | van der Windt, DAWM; van der Heijden, GJMG; van den Berg, SGM; ter Riet, G; de Winter, AF; Bouter, LM | PAIN | 1999 Jun;81(3):257-271. |  |
| 28 | Autologous blood injections for refractory lateral epicondylitis | 9.2 | 174 | Edwards, SG; Calandruccio, JH | J HAND SURG-AM | 2003 Mar;28(2):272-8. |  |
| 29 | Epicondylitis: Pathogenesis, Imaging, and Treatment | 8.9 | 107 | Walz, DM; Newman, JS; Konin, GP; Ross, G | RADIOGRAPHICS | 2010 Jan;30(1):167-84. |  |
| 30 | Ultrasound-guided autologous blood injection for tennis elbow | 8.8 | 141 | Connell, DA; Ali, KE; Ahmad, M; Lambert, S; Corbett, S; Curtis, M | SKELETAL RADIOL | 2006 Jun;35(6):371-7. |  |
| 31 | Analgesic effect of extracorporeal shock-wave therapy on chronic tennis elbow | 8.8 | 228 | Rompe, JD; Hopf, C; Kullmer, K; Heine, J; Burger, R | J BONE JOINT SURG BR | 1996 Mar;78(2):233-7. |  |
| 32 | Interobserver reproducibility of the assessment of severity of complaints, grip strength, and pressure pain threshold in patients with lateral epicondylitis | 8.8 | 175 | Smidt, N; van der Windt, DA; Assendelft, WJ; Mourits, AJ; Deville, WL; de Winter, AF; Bouter, LM | ARCH PHYS MED REHAB | 2002 Aug;83(8):1145-50. |  |
| 33 | The initial effects of a cervical spine manipulative physiotherapy treatment on the pain and dysfunction of lateral epicondylalgia | 8.6 | 224 | Vicenzino, B; Collins, D; Wright, A | PAIN | 1996 Nov;68(1):69-74. |  |
| 34 | Efficacy and safety of steroid injections for shoulder and elbow tendonitis: a meta-analysis of randomised controlled trials | 8.3 | 108 | Gaujoux-Viala, C; Dougados, M; Gossec, L | ANN RHEUM DIS | 2009 Dec;68(12):1843-9. |  |
| 35 | A systematic review of four injection therapies for lateral epicondylosis: prolotherapy, polidocanol, whole blood and platelet-rich plasma | 8.2 | 107 | Rabago, D; Best, TM; Zgierska, AE; Zeisig, E; Ryan, M; Crane, D | BRIT J SPORT MED | 2009 Jul;43(7):471-81. |  |
| 36 | Corticosteroid injections for lateral epicondylitis: a systematic review | 8.1 | 162 | Smidt, N; Assendelft, WJJ; van der Windt, DAWM; Hay, EM; Buchbinder, R; Bouter, LM | PAIN | 2002 Mar;96(1-2):23-40. |  |
| 37 | Hypoalgesic and sympathoexcitatory effects of mobilization with movement for lateral epicondylalgia | 8.0 | 152 | Paungmali, A; O'Leary, S; Souvlis, T; Vicenzino, B | PHYS THER | 2003 Apr;83(4):374-83. |  |
| 38 | Lateral epicondylitis of the elbow: US findings | 7.9 | 134 | Levin, D; Nazarian, LN; Miller, TT; O'Kane, PL; Feld, RI; Parker, L; McShane, JM | RADIOLOGY | 2005 Oct;237(1):230-4. |  |
| 39 | Comparison of Autologous Blood, Corticosteroid, and Saline Injection in the Treatment of Lateral Epicondylitis: A Prospective, Randomized, Controlled Multicenter Study | 7.7 | 85 | Wolf, JM; Ozer, K; Scott, F; Gordon, MJV; Williams, AE | J HAND SURG-AM | 2011 Aug;36(8):1269-72. |  |
| 40 | Pragmatic randomised controlled trial of local corticosteroid injection and naproxen for treatment of lateral epicondylitis of elbow in primary care | 7.6 | 174 | Hay, EM; Paterson, SM; Lewis, M; Hosie, G; Croft, P | BRIT MED J | 1999 Oct 9;319(7215):964-8. |  |
| 41 | Occupation and epicondylitis: a population-based study | 7.5 | 75 | Walker-Bone, K; Palmer, KT; Reading, I; Coggon, D; Cooper, C | RHEUMATOLOGY | 2012 Feb;51(2):305-10. |  |
| 42 | A new integrative model of lateral epicondylalgia | 7.4 | 96 | Coombes, BK; Bisset, L; Vicenzino, B | BRIT J SPORT MED | 2009 Apr;43(4):252-8. |  |
| 43 | Use of extracorporeal shock waves in the treatment of pseudarthrosis, tendinopathy and other orthopedic diseases | 7.3 | 183 | Haupt, G | J UROLOGY | 1997 Jul;158(1):4-11. |  |
| 44 | The efficacy of prolotherapy for lateral epicondylosis: A pilot study | 7.3 | 102 | Scarpone, M; Rabago, DP; Zgierska, A; Arbogast, G; Snell, E | CLIN J SPORT MED | 2008 May;18(3):248-54. |  |
| 45 | Lateral and medial epicondylitis: Role of occupational factors | 7.2 | 79 | Shiri, R; Viikari-Juntura, E | BEST PRACT RES CL RH | 2011 Feb;25(1):43-57. |  |
| 46 | Elbow tendinopathy: tennis elbow | 7.2 | 136 | Nirschl, RP; Ashman, ES | CLIN SPORT MED | 2003 Oct;22(4):813-36. |  |
| 47 | An isokinetic eccentric programme for the management of chronic lateral epicondylar tendinopathy | 7.0 | 105 | Croisier, JL; Foidart-Dessalle, M; Tinant, F; Crielaard, JM; Forthomme, B | BRIT J SPORT MED | 2007 Apr;41(4):269-75. |  |
| 48 | MICROSCOPIC HISTOPATHOLOGY OF CHRONIC REFRACTORY LATERAL EPICONDYLITIS | 6.9 | 207 | REGAN, W; WOLD, LE; COONRAD, R; MORREY, BF | AM J SPORT MED | 1992 Nov-Dec;20(6):746-9. |  |
| 49 | Treatment of lateral epicondylitis using skin-derived tenocyte-like cells | 6.8 | 89 | Connell, D; Datir, A; Alyas, F; Curtis, M | BRIT J SPORT MED | 2009 Apr;43(4):293-8. |  |
| 50 | LATERAL EPICONDYLITIS - CORRELATION OF MR-IMAGING, SURGICAL, AND HISTOPATHOLOGIC FINDINGS | 6.7 | 180 | POTTER, HG; HANNAFIN, JA; MORWESSEL, RM; DICARLO, EF; OBRIEN, SJ; ALTCHEK, DW | RADIOLOGY | 1995 Jul;196(1):43-6. |  |
| 51 | Lateral epicondylitis: Review and current concepts | 6.7 | 100 | Faro, F; Wolf, JM | J HAND SURG-AM | 2007 Oct;32(8):1271-9. |  |
| 52 | Sonographic examination of lateral epicondylitis | 6.5 | 137 | Connell, D; Burke, F; Coombes, P; McNealy, S; Freeman, D; Pryde, D; Hoy, G | AM J ROENTGENOL | 2001 Mar;176(3):777-82. |  |
| 53 | IMMEDIATE HYPOALGESIC AND MOTOR EFFECTS AFTER A SINGLE CERVICAL SPINE MANIPULATION IN SUBJECTS WITH LATERAL EPICONDYLALGIA | 6.4 | 90 | Fernandez-Camero, J; Fernandez-de-las-Penas, C; Cleland, JA | J MANIP PHYSIOL THER | 2008 Nov-Dec;31(9):675-81. |  |
| 54 | Arthroscopic classification and treatment of lateral epicondylitis: Two-year clinical results | 6.4 | 141 | Baker, CL; Murphy, KP; Gottlob, CA; Curd, DT | J SHOULDER ELB SURG | 2000 Nov-Dec;9(6):475-82. |  |
| 55 | TENNIS ELBOW - ANATOMICAL, EPIDEMIOLOGIC AND THERAPEUTIC ASPECTS | 6.4 | 179 | VERHAAR, JAN | INT ORTHOP | 1994 Oct;18(5):263-7. |  |
| 56 | Extracorporeal shock wave therapy in the treatment of lateral epicondylitis - A randomized multicenter trial | 6.4 | 127 | Haake, M; Konig, IR; Decker, T; Riedel, C; Buch, M; Muller, HH | J BONE JOINT SURG AM | 2002 Nov;84(11):1982-91. |  |
| 57 | Shock wave therapy for lateral elbow pain | 6.1 | 104 | Buchbinder, R; Green, SE; Youd, JM; Assendelft, WJJ; Barnsley, L; Smidt, N | COCHRANE DB SYST REV | 2005 Oct 19;2005(4):CD003524. |  |
| 58 | Topical nitric oxide application in the treatment of chronic extensor tendinosis at the elbow - A randomized, double-blinded, placebo-controlled clinical trial | 6.1 | 116 | Paoloni, JA; Appleyard, RC; Nelson, J; Murrell, GAC | AM J SPORT MED | 2003 Nov-Dec;31(6):915-20. |  |
| 59 | Management of lateral epicondylitis: Current concepts | 6.1 | 85 | Calfee, RP; Patel, A; DaSilva, MF; Akelman, E | J AM ACAD ORTHOP SUR | 2008 Jan;16(1):19-29. |  |
| 60 | Medial epicondylitis: is ultrasound guided autologous blood injection an effective treatment? | 6.0 | 96 | Suresh, SPS; Ali, KE; Jones, H; Connell, DA | BRIT J SPORT MED | 2006 Nov;40(11):935-9; discussion 939. |  |
| 61 | Treatment of lateral epicondylitis with botulinum toxin. A randomized, double-blind, placebo-controlled trial | 5.9 | 100 | Wong, SM; Hui, ACF; Tong, PY; Poon, DWF; Yu, E; Wong, LKS | ANN INTERN MED | 2005 Dec 6;143(11):793-7. |  |
| 62 | Extracorporeal shock wave therapy without local anesthesia for chronic lateral epicondylitis | 5.8 | 99 | Pettrone, FA; McCall, BR | J BONE JOINT SURG AM | 2005 Jun;87(6):1297-304. |  |
| 63 | ROTATOR CUFF DEGENERATION AND LATERAL EPICONDYLITIS - A COMPARATIVE HISTOLOGICAL STUDY | 5.8 | 163 | CHARD, MD; CAWSTON, TE; RILEY, GP; GRESHAM, GA; HAZLEMAN, BL | ANN RHEUM DIS | 1994 Jan;53(1):30-4. |  |
| 64 | Acupuncture for the alleviation of lateral epicondyle pain: a systematic review | 5.8 | 104 | Trinh, KV; Phillips, SD; Ho, E; Damsma, K | RHEUMATOLOGY | 2004 Sep;43(9):1085-90. |  |
| 65 | Extracorporeal shock wave therapy for lateral epicondylitis - a double blind randomised controlled trial | 5.8 | 115 | Speed, CA; Nichols, D; Richards, C; Humphreys, H; Wies, JT; Burnet, S; Hazleman, BL | J ORTHOPAED RES | 2002 Sep;20(5):895-8. |  |
| 66 | Lateral tennis elbow: Is there any science out there? | 5.7 | 130 | Boyer, MI; Hastings, H | J SHOULDER ELB SURG | 1999 Sep-Oct;8(5):481-91. |  |
| 67 | Lateral epicondylitis in general practice: Course and prognostic indicators of outcome | 5.6 | 89 | Smidt, N; Lewis, M; Van der Windt, DAMM; Hay, EM; Bouter, LM; Croft, P | J RHEUMATOL | 2006 Oct;33(10):2053-59. |  |
| 68 | Effectiveness of physiotherapy for lateral epicondylitis: a systematic review | 5.5 | 105 | Smidt, N; Assendelft, WJJ; Arola, H; Malmivaara, A; Green, S; Buchbinder, R; van der Windt, DAWM; Bouter, LM | ANN MED | 2003;35(1):51-62. |  |
| 69 | Lateral epicondylitis in tennis: update on aetiology, biomechanics and treatment | 5.5 | 82 | De Smedt, T; de Jong, A; Van Leemput, W; Lieven, D; Van Glabbeek, F | BRIT J SPORT MED | 2007 Nov;41(11):816-9. |  |
| 70 | Treatment of chronic radial epicondylitis with botulinum toxin A - A double-blind, placebo-controlled, randomized multicenter study | 5.5 | 82 | Placzek, R; Drescher, W; Deuretzbacher, G; Hempfing, A; Meiss, AL | J BONE JOINT SURG AM | 2007 Feb;89(2):255-60. |  |
| 71 | Comparison of sonography and MRI for diagnosing epicondylitis | 5.5 | 109 | Miller, TT; Shapiro, MA; Schultz, E; Kalish, PE | J CLIN ULTRASOUND | 2002 May;30(4):193-202. |  |
| 72 | In vivo investigation of ECRB tendons with microdialysis technique - no signs of inflammation but high amounts of glutamate in tennis elbow | 5.3 | 117 | Alfredson, H; Ljung, BO; Thorsen, K; Lorentzon, R | ACTA ORTHOP SCAND | 2000 Oct;71(5):475-9. |  |
| 73 | Physical and psychosocial risk factors for lateral epicondylitis: a population based case-referent study | 5.3 | 100 | Haahr, JP; Andersen, JH | OCCUP ENVIRON MED | 2003 May;60(5):322-9. |  |
| 74 | Corticosteroid injections for lateral epicondylitis: A systematic overview | 5.2 | 135 | Assendelft, WJJ; Hay, EM; Adshead, R; Bouter, LM | BRIT J GEN PRACT | 1996 Apr;46(405):209-16. |  |
| 75 | Tendinosis of the extensor carpi radialis brevis: An evaluation of three methods of operative treatment | 5.1 | 81 | Szabo, SJ; Savoie, FH; Field, LD; Ramsey, JR; Hosemann, CD | J SHOULDER ELB SURG | 2006 Nov-Dec;15(6):721-7. |  |
| 76 | Sensory and motor effects of experimental muscle pain in patients with lateral epicondylalgia and controls with delayed onset muscle soreness | 5.1 | 86 | Slater, H; Arendt-Nielsen, L; Wright, A; Graven-Nielsen, T | PAIN | 2005 Mar;114(1-2):118-30. |  |
| 77 | Conservative treatment of lateral epicondylitis - Brace versus physical therapy or a combination of both - A randomized clinical trial | 5.1 | 91 | Struijs, PAA; Kerkhoffs, GMMJ; Assendelft, WJJ; van Dijk, CN | AM J SPORT MED | 2004 Mar;32(2):462-9. |  |
| 78 | Repetitive low-energy shock wave treatment for chronic lateral epicondylitis in tennis players | 4.9 | 89 | Rompe, JD; Decking, J; Schoellner, C; Theis, C | AM J SPORT MED | 2004 Apr-May;32(3):734-43. |  |
| 79 | LATERAL EXTENSOR RELEASE FOR TENNIS ELBOW - A PROSPECTIVE LONG-TERM FOLLOW-UP-STUDY | 4.9 | 142 | VERHAAR, J; WALENKAMP, G; KESTER, A; VANMAMEREN, H; VANDERLINDEN, T | J BONE JOINT SURG AM | 1993 Jul;75(7):1034-43. |  |
| 80 | Sonographically guided percutaneous needle tenotomy for treatment of common extensor tendinosis in the elbow | 4.7 | 75 | McShane, JM; Nazarian, LN; Harwood, MI | J ULTRAS MED | 2006 Oct;25(10):1281-9. |  |
| 81 | Neurokinin 1-receptors and sensory neuropeptides in tendon insertions at the medial and lateral epicondyles of the humerus - Studies on tennis elbow and medial epicondylalgia | 4.6 | 83 | Ljung, BO; Alfredson, H; Forsgren, S | J ORTHOPAED RES | 2004 Mar;22(2):321-7. |  |
| 82 | Iontophoretic administration of dexamethasone sodium phosphate for acute epicondylitis - A randomized, double-blinded, placebo-controlled study | 4.5 | 86 | Nirschl, RP; Rodin, DM; Ochiai, DH; Maartmann-Moe, C | AM J SPORT MED | 2003 Mar-Apr;31(2):189-95. |  |
| 83 | Botulinum toxin injection in the treatment of tennis elbow - A double-blind, randomized, controlled, pilot study | 4.5 | 76 | Hayton, MJ; Santini, AJA; Hughes, PJ; Frostick, SP; Trail, IA; Stanley, JK | J BONE JOINT SURG AM | 2005 Mar;87(3):503-7. |  |
| 84 | Arthroscopic release for lateral epicondylitis | 4.4 | 93 | Owens, BD; Murphy, KP; Kuklo, TR | ARTHROSCOPY | 2001 Jul;17(6):582-7. |  |
| 85 | Prognostic factors in lateral epicondylitis: a randomized trial with one-year follow-up in 266 new cases treated with minimal occupational intervention or the usual approach in general practice | 4.4 | 84 | Haahr, JP; Andersen, JH | RHEUMATOLOGY | 2003 Oct;42(10):1216-25. |  |
| 86 | Experimental deep tissue pain in wrist extensors - a model of lateral epicondylalgia | 4.1 | 78 | Slater, H; Arendt-Nielsen, L; Wright, A; Graven-Nielsen, T | EUR J PAIN | 2003;7(3):277-88. |  |
| 87 | Local corticosteroid injection versus cyriax-type physiotherapy for tennis elbow | 4.0 | 104 | Verhaar, JAN; Walenkamp, GHIM; vanMameren, H; Kester, ADM; vanderLinden, AJ | J BONE JOINT SURG BR | 1996 Jan;78(1):128-32. |  |
| 88 | Substance P and calcitonin gene-related peptide expression at the extensor carpi radialis brevis muscle origin: Implications for the etiology of tennis elbow | 4.0 | 92 | Ljung, BO; Forsgren, S; Friden, J | J ORTHOPAED RES | 1999 Jul;17(4):554-9. |  |
| 89 | Non-operative treatment regime including eccentric training for lateral humeral epicondylalgia | 4.0 | 83 | Svernlov, B; Adolfsson, L | SCAND J MED SCI SPOR | 2001 Dec;11(6):328-34. |  |
| 90 | Local injection treatment for lateral epicondylitis | 3.8 | 76 | Altay, T; Gunal, I; Ozturk, H | CLIN ORTHOP RELAT R | 2002 May;(398):127-30. |  |
| 91 | Corticosteroid injection in early treatment of lateral epicondylitis | 3.7 | 78 | Newcomer, KL; Laskowski, ER; Idank, DM; McLean, TJ; Egan, KS | CLIN J SPORT MED | 2001 Oct;11(4):214-22. |  |
| 92 | Understanding prognosis to improve rehabilitation: The example of lateral elbow pain | 3.6 | 93 | Hudak, PL; Cole, DC; Haines, AT | ARCH PHYS MED REHAB | 1996 Jun;77(6):586-93. |  |
| 93 | Wrist extensor muscle pathology in lateral epicondylitis | 3.5 | 81 | Ljung, BO; Lieber, RL; Friden, J | J HAND SURG-BRIT EUR | 1999 Apr;24(2):177-83. |  |
| 94 | MR imaging findings of lateral ulnar collateral ligament abnormalities in patients with lateral epicondylitis | 3.4 | 78 | Bredella, MA; Tirman, PFJ; Fritz, RC; Feller, JF; Wischer, TK; Genant, HK | AM J ROENTGENOL | 1999 Nov;173(5):1379-82. |  |
| 95 | Low-energy extracorporal shock wave therapy for persistent tennis elbow | 3.3 | 86 | Rompe, JD; Hopf, C; Kullmer, K; Heine, J; Burger, R; Nafe, B | INT ORTHOP | 1996;20(1):23-7. |  |
| 96 | Epicondylitis among cooks in nursery schools | 3.3 | 78 | Ono, Y; Nakamura, R; Shimaoka, M; Hiruta, S; Hattori, Y; Ichihara, G; Kamijima, M; Takeuchi, Y | OCCUP ENVIRON MED | 1998 Mar;55(3):172-9. |  |
| 97 | LOCAL INJECTION TREATMENT OF TENNIS ELBOW - HYDROCORTISONE, TRIAMCINOLONE AND LIGNOCAINE COMPARED | 2.9 | 90 | PRICE, R; SINCLAIR, H; HEINRICH, I; GIBSON, T | BRIT J RHEUMATOL | 1991 Feb;30(1):39-44. |  |
| 98 | PREVALENCE OF EPICONDYLITIS AND ELBOW PAIN IN THE MEAT-PROCESSING INDUSTRY | 2.8 | 88 | VIIKARIJUNTURA, E; KURPPA, K; KUOSMA, E; HUUSKONEN, M; KUORINKA, I; KETOLA, R; KONNI, U | SCAND J WORK ENV HEA | 1991 Feb;17(1):38-45. |  |
| 99 | THE PREVALENCE AND CAUSATION OF TENNIS ELBOW (LATERAL HUMERAL EPICONDYLITIS) IN A POPULATION OF WORKERS IN AN ENGINEERING INDUSTRY | 2.5 | 88 | DIMBERG, L | ERGONOMICS | 1987 Mar;30(3):573-9. |  |
| 100 | LATERAL HUMERAL EPICONDYLITIS - A STUDY OF NATURAL-HISTORY AND THE EFFECT OF CONSERVATIVE THERAPY | 2.2 | 87 | BINDER, AI; HAZLEMAN, BL | BRIT J RHEUMATOL | 1983 May;22(2):73-6. |  |
