# Supplementary material for: The 100 most cited articles in lateral epicondylitis research: A bibliometric analysis
Source: Front Surg. 2023 Feb 13;9:913818. doi: 10.3389/fsurg.2022.913818 (PMC9968860; doi:10.3389/fsurg.2022.913818)
Supplement: Supplementary file 2 [file Table2.docx]

| Table 2. The journal to which 100 Cited Articles on Tennis elbow | | | | | | | |
| --- | --- | --- | --- | --- | --- | --- | --- |
| Journal | **N** | **Total Citation** | **Mean Citation** | **IF** | **total Citation density** | **mean Citation density** | **mean Citation density rank** |
| AMERICAN JOURNAL OF SPORTS MEDICINE | 13 | 2550 | 196.2 | 6.202 | 204.8 | 15.8 | 5 |
| BRITISH JOURNAL OF SPORTS MEDICINE | 10 | 1190 | 119.0 | 13.8 | 89.9 | 9.0 | 10 |
| JOURNAL OF BONE AND JOINT SURGERY-AMERICAN VOLUME | 7 | 1392 | 198.9 | 5.284 | 55.4 | 7.9 | 15 |
| PAIN | 4 | 686 | 171.5 | 6.961 | 31.1 | 7.8 | 17 |
| RHEUMATOLOGY | 4 | 384 | 96.0 | 7.58 | 27.0 | 6.8 | 24 |
| JOURNAL OF HAND SURGERY-AMERICAN VOLUME | 3 | 359 | 119.7 | 2.23 | 23.6 | 7.9 | 16 |
| AMERICAN JOURNAL OF ROENTGENOLOGY | 3 | 368 | 122.7 | 3.959 | 21.7 | 7.2 | 21 |
| JOURNAL OF SHOULDER AND ELBOW SURGERY | 3 | 352 | 117.3 | 3.019 | 17.1 | 5.7 | 31 |
| JOURNAL OF ORTHOPAEDIC RESEARCH | 3 | 290 | 96.7 | 3.494 | 14.4 | 4.8 | 39 |
| LANCET | 2 | 852 | 426.0 | 79.321 | 57.6 | 28.8 | 1 |
| CLINICS IN SPORTS MEDICINE | 2 | 425 | 212.5 | 2.182 | 16.8 | 8.4 | 13 |
| RADIOLOGY | 2 | 314 | 157.0 | 11.105 | 14.5 | 7.3 | 20 |
| ANNALS OF THE RHEUMATIC DISEASES | 2 | 271 | 135.5 | 19.013 | 14.1 | 7.1 | 23 |
| JOURNAL OF BONE AND JOINT SURGERY-BRITISH VOLUME | 2 | 332 | 166.0 | / | 12.8 | 6.4 | 25 |
| ARCHIVES OF PHYSICAL MEDICINE AND REHABILITATION | 2 | 268 | 134.0 | 3.966 | 12.3 | 6.2 | 27 |
| CLINICAL JOURNAL OF SPORT MEDICINE | 2 | 180 | 90.0 | 3.638 | 11.0 | 5.5 | 34 |
| INTERNATIONAL ORTHOPAEDICS | 2 | 265 | 132.5 | 3.075 | 9.7 | 4.9 | 38 |
| OCCUPATIONAL AND ENVIRONMENTAL MEDICINE | 2 | 178 | 89.0 | 4.402 | 8.5 | 4.3 | 42 |
| BRITISH JOURNAL OF RHEUMATOLOGY | 2 | 177 | 88.5 | / | 5.1 | 2.6 | 48 |
| AMERICAN JOURNAL OF EPIDEMIOLOGY | 1 | 345 | 345.0 | 4.897 | 21.6 | 21.6 | 2 |
| JAMA-JOURNAL OF THE AMERICAN MEDICAL ASSOCIATION | 1 | 168 | 168.0 | 56.272 | 18.7 | 18.7 | 3 |
| BMJ-BRITISH MEDICAL JOURNAL | 1 | 269 | 269.0 | 39.89 | 16.8 | 16.8 | 4 |
| BONE & JOINT JOURNAL | 1 | 101 | 101.0 | 5.082 | 11.2 | 11.2 | 6 |
| BMC MUSCULOSKELETAL DISORDERS | 1 | 137 | 137.0 | 2.362 | 9.8 | 9.8 | 7 |
| JOURNAL OF HAND THERAPY | 1 | 144 | 144.0 | 1.95 | 9.6 | 9.6 | 8 |
| CLINICAL JOURNAL OF PAIN | 1 | 122 | 122.0 | 3.442 | 9.4 | 9.4 | 9 |
| RADIOGRAPHICS | 1 | 107 | 107.0 | 5.333 | 8.9 | 8.9 | 11 |
| SKELETAL RADIOLOGY | 1 | 141 | 141.0 | 2.199 | 8.8 | 8.8 | 12 |
| PHYSICAL THERAPY | 1 | 152 | 152.0 | 3.021 | 8.0 | 8.0 | 14 |
| BRITISH MEDICAL JOURNAL | 1 | 174 | 174.0 | / | 7.6 | 7.6 | 18 |
| JOURNAL OF UROLOGY | 1 | 183 | 183.0 | 7.45 | 7.3 | 7.3 | 19 |
| BEST PRACTICE & RESEARCH IN CLINICAL RHEUMATOLOGY | 1 | 79 | 79.0 | 4.098 | 7.2 | 7.2 | 22 |
| JOURNAL OF MANIPULATIVE AND PHYSIOLOGICAL THERAPEUTICS | 1 | 90 | 90.0 | 1.437 | 6.4 | 6.4 | 26 |
| COCHRANE DATABASE OF SYSTEMATIC REVIEWS | 1 | 104 | 104.0 | 9.266 | 6.1 | 6.1 | 28 |
| JOURNAL OF THE AMERICAN ACADEMY OF ORTHOPAEDIC SURGEONS | 1 | 85 | 85.0 | 3.02 | 6.1 | 6.1 | 29 |
| ANNALS OF INTERNAL MEDICINE | 1 | 100 | 100.0 | 25.391 | 5.9 | 5.9 | 30 |
| JOURNAL OF RHEUMATOLOGY | 1 | 89 | 89.0 | 4.666 | 5.6 | 5.6 | 32 |
| ANNALS OF MEDICINE | 1 | 105 | 105.0 | 4.709 | 5.5 | 5.5 | 33 |
| JOURNAL OF CLINICAL ULTRASOUND | 1 | 109 | 109.0 | 0.91 | 5.5 | 5.5 | 35 |
| ACTA ORTHOPAEDICA SCANDINAVICA | 1 | 117 | 117.0 | 3.7 | 5.3 | 5.3 | 36 |
| BRITISH JOURNAL OF GENERAL PRACTICE | 1 | 135 | 135.0 | 5.386 | 5.2 | 5.2 | 37 |
| JOURNAL OF ULTRASOUND IN MEDICINE | 1 | 75 | 75.0 | 2.153 | 4.7 | 4.7 | 40 |
| ARTHROSCOPY-THE JOURNAL OF ARTHROSCOPIC AND RELATED SURGERY | 1 | 93 | 93.0 | 4.772 | 4.4 | 4.4 | 41 |
| EUROPEAN JOURNAL OF PAIN | 1 | 78 | 78.0 | 3.931 | 4.1 | 4.1 | 43 |
| SCANDINAVIAN JOURNAL OF MEDICINE & SCIENCE IN SPORTS | 1 | 83 | 83.0 | 4.221 | 4.0 | 4.0 | 44 |
| CLINICAL ORTHOPAEDICS AND RELATED RESEARCH | 1 | 76 | 76.0 | 4.176 | 3.8 | 3.8 | 45 |
| JOURNAL OF HAND SURGERY-BRITISH AND EUROPEAN VOLUME | 1 | 81 | 81.0 | / | 3.5 | 3.5 | 46 |
| SCANDINAVIAN JOURNAL OF WORK ENVIRONMENT & HEALTH | 1 | 88 | 88.0 | 5.024 | 2.8 | 2.8 | 47 |
| ERGONOMICS | 1 | 88 | 88.0 | 2.778 | 2.5 | 2.5 | 49 |
